# Fabrication of NiFe-LDHs Modified Carbon Nanotubes as the High-Performance Sulfur Host for Lithium–Sulfur Batteries

**DOI:** 10.3390/nano14030272

**Published:** 2024-01-27

**Authors:** Lingwei Zhang, Runlan Li, Wenbo Yue

**Affiliations:** 1 Beijing Key Laboratory of Energy Conversion and Storage Materials, College of Chemistry, Beijing Normal University, Beijing 100875, China; 2215015049@stu.xjtu.edu.cn (L.Z.); 201731150008@mail.bnu.edu.cn (R.L.); 2 College of Chemistry, Xi’an Jiaotong University, Xi’an 710049, China

**Keywords:** NiFe-LDHs, CNT, sulfur host, shuttle effect, Li-S battery

## Abstract

Lithium–sulfur batteries offer the potential for significantly higher energy density and cost-effectiveness. However, their progress has been hindered by challenges such as the “shuttle effect” caused by lithium polysulfides and the volume expansion of sulfur during the lithiation process. These limitations have impeded the widespread adoption of lithium–sulfur batteries in various applications. It is urgent to explore the high-performance sulfur host to improve the electrochemical performance of the sulfur electrode. Herein, bimetallic NiFe hydroxide (NiFe-LDH)-modified carbon nanotubes (CNTs) are prepared as the sulfur host materials (NiFe-CNT@S) for loading of sulfur. On the one hand, the crosslinked CNTs can increase the electron conductivity of the sulfur host as well as disperse NiFe-LDHs nanosheets. On the other hand, NiFe-LDHs command the capability of strongly adsorbing lithium polysulfides and also accelerate their conversion, which effectively suppresses the shuttle effect problem in lithium polysulfides. Hence, the electrochemical properties of NiFe-CNT@S exhibit significant enhancements when compared with those of the sulfur-supported pure NiFe-LDHs (NiFe-LDH@S). The initial capacity of NiFe-CNT@S is reported to be 1010 mAh g^−1^. This value represents the maximum amount of charge that the material can store per gram when it is first synthesized or used in a battery. After undergoing 500 cycles at a rate of 2 C (1 C = 1675 mA g^−1^), the NiFe-CNT@S composite demonstrates a sustained capacity of 876 mAh g^−1^. Capacity retention is a measure of how well a battery or electrode material can maintain its capacity over repeated charge–discharge cycles, and a higher retention percentage indicates better durability and stability of the material.

## 1. Introduction

Although there is still sufficient fossil energy worldwide, it is urgent to achieve a breakthrough in the fields of science and technology. As a major advancement in new energy development, secondary batteries can effectively address various issues such as multiple energy utilization and sustainable development. Lithium–sulfur batteries, ranked second among batteries due to their excellent performance, boast a high energy density of 2600 Wh kg^−1^, making them highly significant [1]. The specific energy density of lithium–sulfur batteries is nearly five times higher than that of conventional lithium batteries. However, the progress of lithium–sulfur battery technology has been limited by technical constraints such as the dissolution of polysulfides, the shuttle effect resulting from migration, and the growth of lithium dendrites [2,3,4]. Additionally, the large volume change and poor electron conductivity of sulfur during the lithiation process also lead to performance degradation [5,6]. In order to address these problems, scientists have discovered an effective method of combining conductive materials with sulfur to enhance electrode conductivity and reduce the shuttle effect of polysulfides [7,8]. Various carbon materials, including heterocarbon, graphene oxide, and functional-group-containing carbon nanotubes/nanofibers, have been studied as sulfur hosts for lithium–sulfur batteries and have shown certain effects in preventing the shuttle effect of lithium polysulfides [9,10,11]. However, carbon materials typically have non-polar surfaces and weak physical adsorption capacity for polar lithium polysulfides. Therefore, it is still urgent for us to identify new materials that can efficiently adsorb lithium polysulfides and accelerate their conversion.

Currently, it has been found that modifying carbon materials with metal compounds can effectively enhance their ability to capture lithium polysulfides. For example, CeO_2_-modified CMK-3 is considered as a sulfur host for lithium–sulfur batteries. The mesoporous structure of CMK-3 provides the necessary space for sulfur volume expansion. Furthermore, the CeO_2_ nanoparticles can transform the non-polar carbon surface into a polar surface, which can strongly capture lithium polysulfides and effectively suppress the shuttle effect [12]. Another notable example in the realm of lithium–sulfur batteries is the incorporation of a novel self-supported interlayer (Co_4_S_3_/C@CC). This interlayer is developed by introducing carbon cloth (CC) with Co_4_S_3_-embedded carbon nanoarrays, displaying high potential for enhancing battery performance. The Co_4_S_3_ nanoparticles in the interlayer not only effectively inhibit the shuttle effect of polysulfides but also significantly promote lithium-ion diffusion. The carbon substrate between the polar Co_4_S_3_ nanoparticles and the conductive material also serves as a reaction site to accelerate polysulfide conversion and guide the flower-like growth of Li_2_S, ultimately alleviating interlayer surface passivation and improving sulfur utilization [13]. Previous research has shown that bimetallic compounds have excellent adsorption capabilities for lithium polysulfides. Layered double metal hydroxides (LDHs) possess a distinctive layered structure comprising positively charged hydroxide host layers and interlayer guest anions [14,15]. The exchangeability of interlayer ions facilitates the introduction of functional guest substances into the interlayer space, such as lithium polysulfides. Additionally, the metals in the main laminate can form M-S bonds (M is metal) to capture lithium polysulfides. Density Functional Theory (DFT) calculation results suggest that there may be a hydrogen bonding interaction between the -OH functional group on the surface of LDHs and the S atoms in lithium polysulfides, further enhancing the effect of LDHs on lithium polysulfide adsorption. Therefore, LDHs are considered excellent sulfur host materials for use in lithium–sulfur batteries.

Herein, a NiFe-LDHs-modified carbon nanotube (NiFe-CNT) was prepared by a hydrothermal method and served as the sulfur host for lithium–sulfur batteries. Sulfur-loaded NiFe-CNT (NiFe-CNT@S) was then prepared using a melt-impregnation method, and its electrochemical properties were also tested. On the one hand, CNTs have a cross-linked network structure that not only provides a conductive network but also facilitates the migration of lithium ions; moreover, the growth of NiFe-LDHs on CNTs can effectively disperse NiFe-LDHs nanosheets and increase the contact area between NiFe-LDHs and lithium polysulfides. On the other hand, NiFe-LDHs exhibit strong adsorption of lithium polysulfides and promote their conversion. Therefore, NiFe-CNT@S exhibits excellent cycle and rate performance. The initial capacity of NiFe-CNT@S is 1010 mAh g^−1^, and the capacity is maintained at 876 mAh g^−1^ with a capacity retention of 86.7% at a rate of 2 C after 500 cycles. This study demonstrates the potential applications of LDHs as high-performance sulfur hosts for lithium–sulfur batteries.

## 2. Experiment

### 2.1. Sample Preparation

#### 2.1.1. Synthesis of NiFe-LDHs

In a typical synthesis of NiFe-LDHs, 1.40 g of nickel nitrate hexahydrate and 0.97 g of ferric nitrate pentahydrate were dissolved in 320 mL of deionized water. Urea was then added to achieve an overall urea content of 35 mmol L^−1^. After thorough stirring for 30 min, 0.0232 g of sodium citrate, serving as a pH modifier, was finally introduced into the solution. Subsequently, the suspension was transferred into a Teflon-lined stainless steel autoclave and heated at 150 °C for 24 h. The resulting product was separated from the supernatant in the upper layer. The remaining product was washed several times with deionized water and ethanol to remove excess ions, and then dried at 60 °C for 12 h in a vacuum drying oven. The product (NiFe-LDHs) was ground into powder for subsequent use.

#### 2.1.2. Preparation of NiFe-CNT

In terms of NiFe-CNT, 0.05 g of carbon nanotubes (CNTs) was dispersed in 160 mL of wastewater using ultrasonic conditions. Then, 0.69 g of nickel nitrate hexahydrate and 0.48 g of ferric nitrate pentahydrate were dissolved in the suspension under stirring. Subsequently, 35 mmol L^−1^ of urea and 0.016 g of sodium citrate were added. After sufficient stirring, the suspension was transferred into a Teflon-lined stainless steel autoclave and heated at 150 °C for 24 h. The product was collected by centrifugation, washed with wastewater and ethanol three times, and dried at 60 °C for 12 h in a vacuum drying oven. The resulting product (NiFe-CNT) was ground into powder for later use.

#### 2.1.3. Preparation of NiFe-LDH@S and NiFe-CNT@S 

NiFe-LDH@S and NiFe-CNT@S were prepared by a traditional melt-impregnation method. In brief, NiFe-LDHs/NiFe-CNT and sulfur were mixed by grinding with a weight ratio of 3:7. The mixture was then heated at 155 °C for 6 h under an Ar atmosphere in a sealed glass bottle. NiFe-LDH@S or NiFe-CNT@S was obtained after the temperature dropped to room temperature. 

### 2.2. Sample Characterization

The samples were characterized using X-ray diffraction (XRD) on a Phillips X’pert Pro MPD diffractometer (Phillips, Amsterdam, The Netherlands), with Cu K_α_ radiation as the light source. The data obtained were analyzed in detail using X’pert HighScore Plus analysis software (v3.0.5) and its database. X-ray photoelectron spectroscopy (XPS) data were obtained using a VG Scientific ESCALab220i-XL electron spectrometer with 300 W Al K_α_ radiation. XPS Peak Fit Software (v4.1) was used to analyze the samples. Thermogravimetric analyzer (TGA) tests (Mettler-Toledo, Zurich, Switzerland) were performed to analyze the sulfur content in the materials. The temperature was gradually increased from 25 °C to the target temperature at a heating rate of 10 °C per minute. The morphology and structure of the samples were observed using a HITACHI S-8010 electron microscope (HITACHI, Tokyo, Japan) under the conditions of 5–10 kV and a 5 mm working distance. Additionally, X-ray energy dispersive spectroscopy (EDX) was equipped under the scanning electron microscope to qualitatively analyze the possible elements and their distribution in the target material. Transmission electron microscopy (TEM) was utilized to observe the morphology and structures of the samples, while the use of high-resolution TEM was deemed necessary. Finally, a FEI Talos F200S electron microscope (Thermo Fisher Scientific, Waltham, MA, USA) with a field acceleration voltage of 200 kV was employed.

### 2.3. Performance Tests

A slurry was prepared by mixing NiFe-LDH@S/NiFe-CNT@S, carbon black, and polyvinylidene fluoride (PVDF) at a weight ratio of 8:1:1 with *N*-methyl-2-pyrrolidone (NMP). The slurry was then coated onto a carbon-coated aluminum current collector. After drying in a vacuum oven at 60 °C for 12 h, a positive electrode was obtained. The negative electrode consisted of lithium foil, and a Celgard 2400 membrane was used as the separator. Coin cells (type 2032) were assembled in an argon-filled glovebox (MBRAUN-UNIlab, Munich, Germany). The electrolyte used was a solution of 1,3-dioxolane (DOL) and 1,2-dimethoxymethane (DME) with a volume ratio of 1:1, containing 1 M lithium bis(trifluoromethanesulfonyl)imide (LiTFSI) and 2 wt% LiNO_3_. Cyclic voltammetry (CV) and electrochemical impedance spectroscopy (EIS) were performed using a Gamry Interface 1000 electrochemical station (Gamry Instruments, Warminster, PA, USA). The CV scan rate was set at 0.1 mV s^−1^ and the EIS test frequency range was 100 kHz–10 MHz. The obtained data were analyzed using ZSimpWin software (https://www.ameteksi.com/products/software/zsimpwin, accessed on 18 January 2024) to determine the resistance values of the corresponding materials. The cycle and rate performance of the samples were evaluated using a LAND CT2001A battery tester at room temperature, with a test voltage range of 1.7–2.8 V. The polysulfides in the supernatant after the adsorption experiment were studied using Shimadzu’s (Kyoto, Japan) UV-3600 product for UV testing. The wavelength range of the test was 200–800 nm and a high scanning speed was selected.

## 3. Results and Discussion

### 3.1. NiFe-CNT@S and NiFe-LDH@S

**Figure 1:**
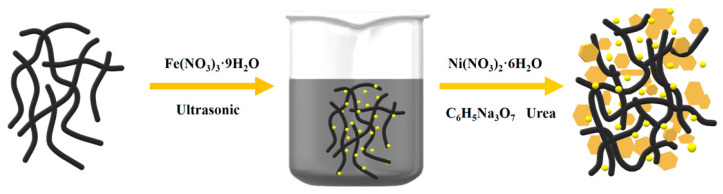
Schematic diagram of the synthesis route for NiFe-CNT.

Carbon nanotubes (CNTs) are extensively employed in various fields such as energy storage and catalysis due to their exceptional electrical conductivity. Consequently, the growth of NiFe-LDHs nanosheets on CNTs presents a promising approach to enhance the conductivity and dispersion of NiFe-LDHs. Additionally, the modification of non-polar CNT surfaces with NiFe-LDHs can convert them into polar surfaces, thereby enhancing the adsorption capacity of lithium polysulfide. Figure 1 illustrates the synthesis route for NiFe-CNT. Initially, carboxylated CNTs are dispersed in a solution containing ferric nitrate by ultrasonication. Fe^3+^ ions are adsorbed on the carboxyl groups of CNTs through electrostatic interaction. Subsequently, nickel nitrate and urea are added to the solution to facilitate the formation of NiFe-LDHs nanosheets on the surface of CNTs. By controlling the concentration of the reaction precursors, NiFe-LDH materials with varying morphologies and particle sizes can be obtained on CNTs. In this study, NiFe-CNT is synthesized under optimized conditions. 

**Figure 2:**
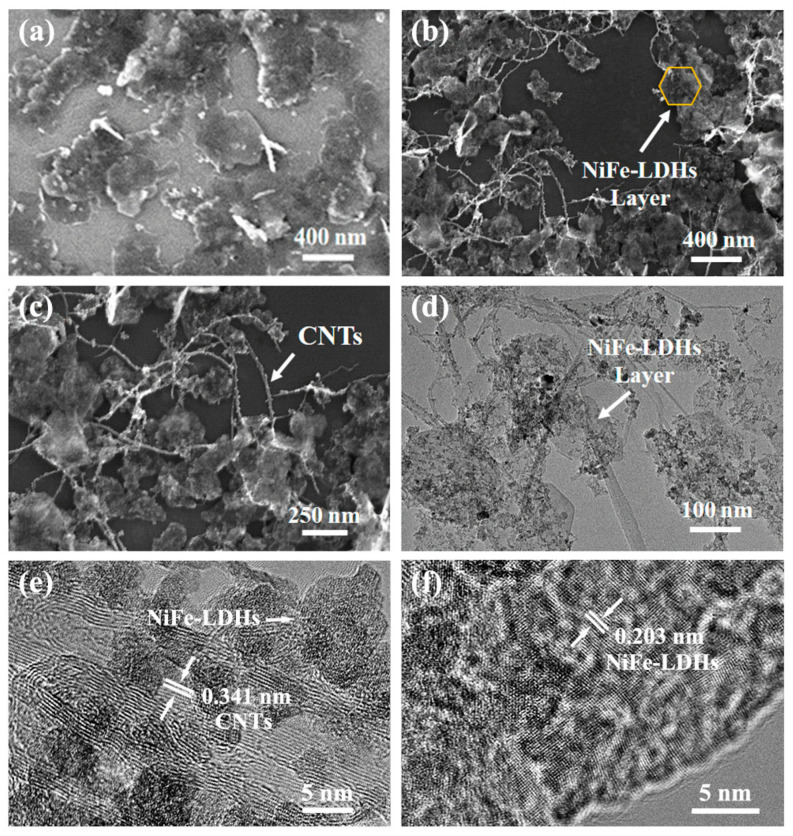
SEM images of (**a**) NiFe-LDHs and (**b**,**c**) NiFe-CNT. (**d**) TEM and (**e**,**f**) HRTEM images of NiFe-CNT.

The morphology and structure of NiFe-LDHs and NiFe-CNT were observed using scanning electron microscopy (SEM). The SEM image of pure NiFe-LDHs (Figure 2a) reveals a 2D nanosheet structure, but the hexagonal structure is not clearly visible. Additionally, the stacking of NiFe-LDH nanosheets is also observed, which is not conducive to the contact between NiFe-LDHs and sulfur. In contrast, the SEM images of NiFe-CNT (Figure 2b,c) demonstrate successful formation of NiFe-LDH nanosheets on the crosslinked CNTs, and the aggregation of NiFe-LDHs is inhibited. The highly dispersed NiFe-LDH nanosheets are favorable for trapping lithium polysulfides. The diameter of NiFe-LDH nanosheets grown on CNTs is approximately 200–300 nm, smaller than that of pure NiFe-LDH nanosheets (>400 nm). The morphology and structure of NiFe-CNT were further examined using transmission electron microscopy (TEM). The TEM image (Figure 2d) clearly shows that NiFe-LDH nanosheets grow at the cross-linking points of CNTs, forming a 3D network structure for sulfur loading. HRTEM image of NiFe-CNT (Figure 2e) reveal lattice fringes with a spacing of 0.341 nm, corresponding to the layers of multi-walled CNTs [16]. Lattice fringes with a spacing of 0.203 nm, which correspond to the (018) plane of the NiFe-LDHs crystal, are also observed in the HRTEM image (Figure 2f), indicating the successful growth of NiFe-LDH nanosheets on CNTs [17]. The surface structures of CNTs before and after growing NiFe-LDHs were further studied by Raman spectroscopy (Figure S1). The peak intensity ratio (*I*_D_/*I*_G_) can reflect the surface defects of CNTs [18]. It is found that the *I*_D_/*I*_G_ value of NiFe-CNT (1.25) is slightly lower than that of CNTs (1.28), indicating that the hydrothermal treatment can improve the reduction degree of CNTs. 

**Figure 3:**
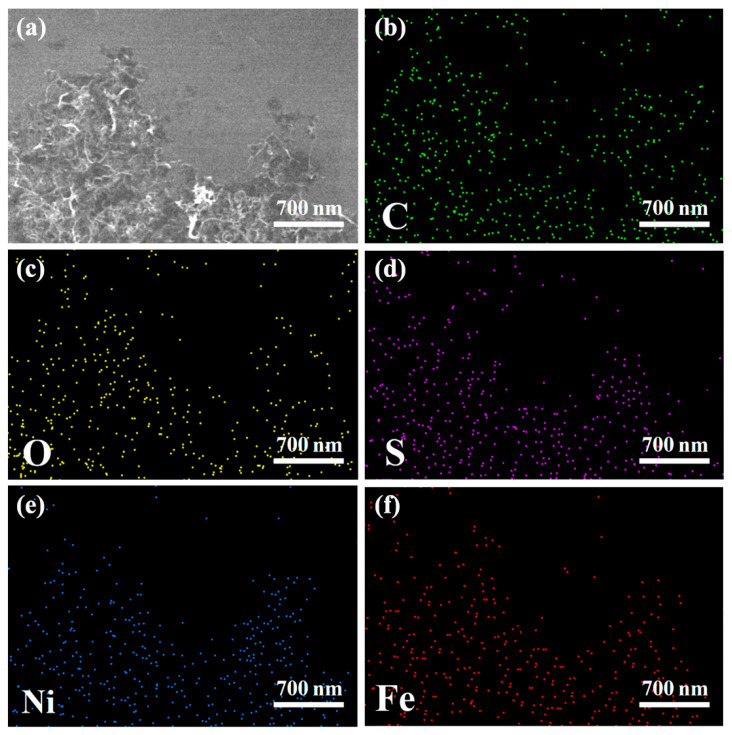
(**a**) SEM image of NiFe-CNT@S and EDX elemental mappings of (**b**) C, (**c**) O, (**d**) S, (**e**) Ni, and (**f**) Fe.

Sulfur was loaded onto NiFe-CNT by a melt-impregnation method. SEM-EDX analysis was conducted on NiFe-CNT@S (Figure 3), revealing that the distribution of the S element is consistent with that of the C, O, Ni, and Fe elements. This indicates the successful loading of sulfur particles onto NiFe-CNT. For comparison, NiFe-LDH@S was also observed by SEM-EDX. The EDX elemental mapping images of NiFe-LDH@S (Figure S2) show evenly distributed O, S, Ni, and Fe elements, proving that the sulfur is well-dispersed on NiFe-LDHs. 

**Figure 4:**
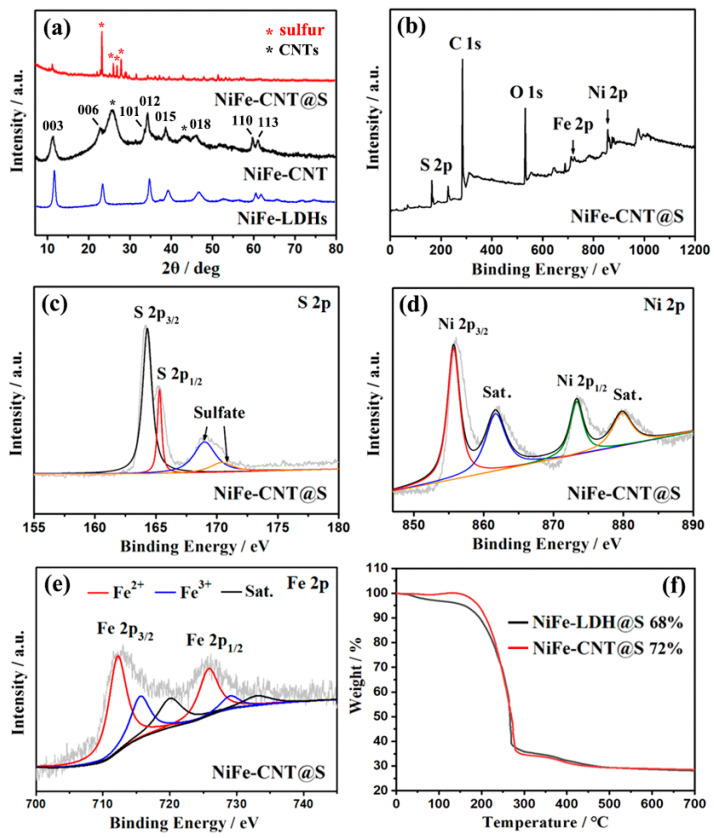
(**a**) XRD patterns of NiFe-LDHs, NiFe-CNT, and NiFe-CNT@S. (**b**) XPS survey spectrum of NiFe-CNT@S. High-resolution (**c**) S 2p, (**d**) Ni 2p, and (**e**) Fe 2p XPS spectra of NiFe-CNT@S. (**f**) TGA curves of NiFe-LDH@S and NiFe-CNT@S.

The crystal structure and composition of NiFe-CNT@S were studied by XRD and XPS. Figure 4a presents the XRD patterns of NiFe-LDHs and NiFe-CNT, with characteristic peaks observed at 11.4°, 22.9°, 34.3°, 38.6°, 46.1°, 59.6°, and 60.9°. These peaks correspond to the (003), (006), (012), (015), (018), (110), and (113) planes of NiFe-LDHs crystals, respectively [19,20]. Additionally, a characteristic peak at 25.6° corresponds to the (002) plane of multi-walled CNTs [21]. After sulfur filling, several new characteristic peaks, belonging to sulfur crystals, appear between 25° and 30° in the XRD pattern of NiFe-CNT@S, indicating successful sulfur loading on NiFe-CNT [22]. In Figure 4b, the XPS survey spectrum of NiFe-CNT@S displays clear characteristic peaks of S 2p, C 1s, O 1s, Fe 2p, and Ni 2p, corresponding to NiFe-LDHs, CNTs, and sulfur particles. Furthermore, the high-resolution S 2p XPS spectrum (Figure 4c) shows two peaks at 164.2 and 165.2 eV, corresponding to the 2p_3/2_ and 2p_1/2_ peaks of S. The peaks at 169.2 and 170.4 eV are related to sulfur oxides such as sulfate or sulfonate, which are often observed in the XPS characterization of sulfur electrode materials [7]. In the high-resolution XPS spectrum of Ni 2p (Figure 4d), the peaks at 855.7 and 873.3 eV correspond to characteristic peaks of Ni 2p_3/2_ and Ni 2p_1/2_ of Ni^2+^, respectively, while the peaks at 861.6 and 879.8 eV are their satellite peaks [23]. Additionally, in the high-resolution XPS spectrum of Fe 2p (Figure 4e), the peaks at 712.2, 725.8, 715.6, and 729.0 eV correspond to Fe^2+^ and Fe^3+^ [24]. The positions of Fe and Ni peaks are consistent with those in the pure NiFe-LDHs reported in our previous work [5], proving the formation of NiFe-LDHs on CNTs. TGA analysis was performed on NiFe-LDH@S and NiFe-CNT@S to evaluate their sulfur contents [25]. According to the TGA curves (Figure 4f), the sulfur content of NiFe-CNT@S is 72 wt%, which is similar to that of NiFe-LDH@S (68 wt%). The similar sulfur loadings of the samples can avoid the influence of different sulfur amounts on the electrochemical properties. The thermal stability of NiFe-CNT and NiFe-LDHs have been discussed in the literature [26]. 

### 3.2. Electrochemical Performance

**Figure 5:**
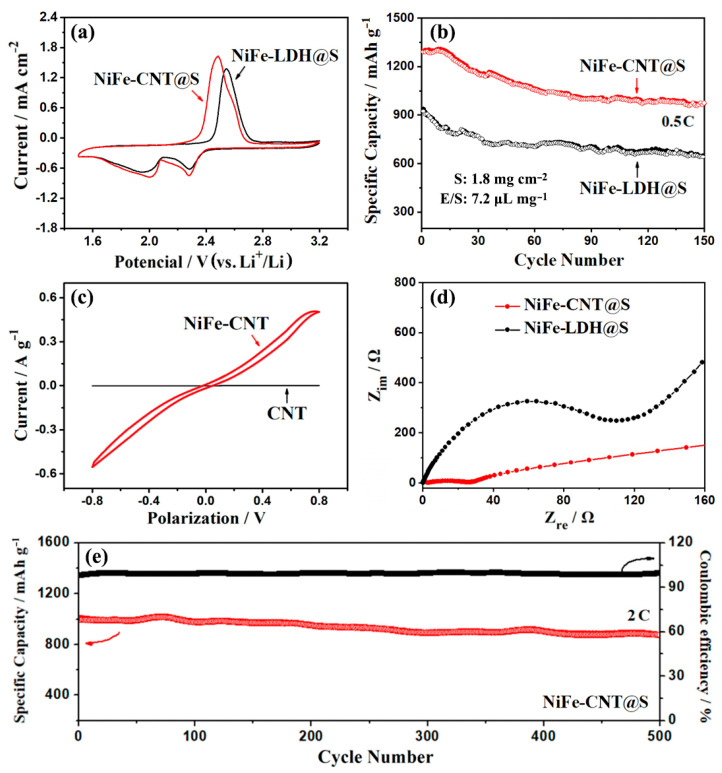
(**a**) Cyclic voltammograms and (**b**) cyclic performance of NiFe-LDH@S and NiFe-CNT@S at 0.5 C. (**c**) CV curves of symmetrical cells assembled with NiFe-CNT and CNT. (**d**) EIS spectra of NiFe-LDH@S and NiFe-CNT@S. (**e**) Long-term cycling performance of NiFe-CNT@S at 2 C.

We compared the performance of NiFe-LDHs and NiFe-CNTs as sulfur hosts by assembling cells with NiFe-LDH@S and NiFe-CNT@S as the positive electrode materials. Figure 5a shows the cyclic voltammograms (CVs) of NiFe-LDH@S and NiFe-CNT@S at a scanning rate of 0.2 mV s^−1^. In the CV curve of NiFe-CNT@S, there are two distinct reduction peaks at 2.28 V and 2.00 V, corresponding to the conversion of S_8_ to long-chain lithium polysulfides and lithium polysulfides to Li_2_S/Li_2_S_2_, respectively. Additionally, an oxidation peak at 2.48 V is observed, representing the oxidation process of Li_2_S/Li_2_S_2_ to S_8_ [27]. In contrast, the reduction peaks and oxidation peak of NiFe-LDH@S appear at 2.28 V, 1.96 V, and 2.54 V, respectively. The voltage difference between the redox peaks of NiFe-CNT@S is 0.20 V, which is smaller than that of NiFe-LDH@S (0.26 V), indicating a lower electrode polarization of NiFe-CNT@S. We conducted cycling performance tests on NiFe-LDH@S and NiFe-CNT@S at 0.5 C for comparison. The initial discharge-specific capacity of NiFe-CNT@S is 1294 mAh g^−1^, which is higher than that of NiFe-LDH@S (937 mAh g^−1^). After 150 cycles, the capacity of NiFe-CNT@S remains at 971 mAh g^−1^, which is still higher than that of NiFe-LDH@S (629 mAh g^−1^). The superior electrochemical performance of NiFe-CNT@S can be attributed to the synergistic effect of NiFe-LDHs and CNTs. The cross-linked CNTs provide a conductive network to improve the electron conductivity of NiFe-CNT@S. Furthermore, the growth of NiFe-LDHs on CNTs inhibits the aggregation of LDH nanosheets and increases their contact area with sulfur, thereby enhancing the adsorption and catalytic ability of NiFe-LDHs for lithium polysulfides.

To verify the catalytic activity of NiFe-LDHs in the conversion of lithium polysulfides, we assembled symmetric cells using NiFe-CNT and pure CNT as the electrodes. As shown in Figure 5c, the cell with CNTs as the symmetric electrodes shows almost no electrochemical response to Li_2_S_6_, indicating that CNTs cannot promote the conversion of lithium polysulfides [28]. In contrast, the cell with NiFe-CNT as the symmetric electrodes exhibits a significant electrochemical response to Li_2_S_6_, indicating that NiFe-LDHs can accelerate the redox reaction of sulfur. 

EIS tests were also performed on NiFe-LDH@S and NiFe-CNT@S. Figure 5d shows the Nyquist plots of the two samples, which display a semicircle in the high-frequency region and an oblique line in the low-frequency region [29]. The diameter of the semicircle represents the charge transfer resistance (*R*_ct_) of the samples. After fitting based on the Randles equivalent circuit (Figure S3), the *R*_ct_ value of NiFe-CNT@S is 25 Ω, much smaller than that of NiFe-LDH@S (106 Ω). This indicates that NiFe-LDH nanosheets are highly dispersed on CNTs and expose more active sites than pure NiFe-LDH nanosheets. Therefore, the redox reaction of sulfur on NiFe-CNT is faster than that on NiFe-LDHs. 

The long-term cycling performance of NiFe-CNT@S is shown in Figure 5e. The initial capacity of NiFe-CNT@S is 1010 mAh g^−1^ at 2 C, and the capacity is maintained at 876 mAh g^−1^ after 500 cycles with a capacity retention of 86.7%. The performance comparison of NiFe-CNT@S with other LDH-related materials is listed in Table 1, where NiFe-CNT@S demonstrates competitive performance. 

**Figure 6:**
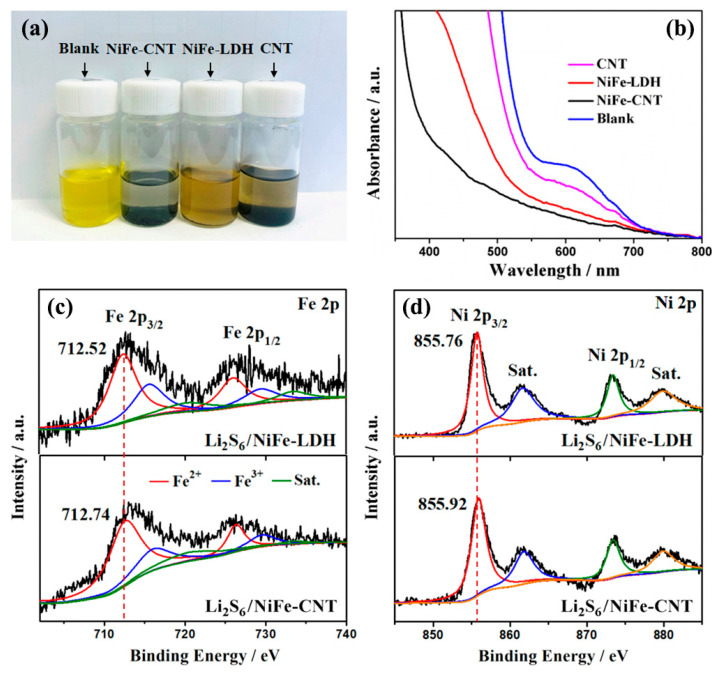
(**a**) Digital photos of Li_2_S_6_ solutions with addition of NiFe-CNT, NiFe-LDHs, and CNT; (**b**) UV–Vis spectra of these solutions. High-resolution XPS spectra of (**c**) Fe 2p and (**d**) Ni 2p for NiFe-LDH and NiFe-CNT after adsorption of lithium polysulfide.

The adsorption capacity of NiFe-LDHs for lithium polysulfides was also investigated. Adsorption experiments were conducted by adding NiFe-CNT, NiFe-LDHs, and CNTs to a Li_2_S_6_ solution. The results after 6 h of adsorption are shown in Figure 6a. It is evident that NiFe-CNT exhibits the highest adsorption performance for lithium polysulfides, followed by NiFe-LDHs, while CNTs show the lowest adsorption performance. UV–Vis spectra of these three samples (Figure 6b) also confirm these findings. The peak corresponding to Li_2_S_6_ (600–650 nm) gradually weakens, indicating that the order of adsorption performance is NiFe-CNT > NiFe-LDHs > CNTs. The Li_2_S_6_ adsorbed on NiFe-CNT (Li_2_S_6_/NiFe-CNT) was collected and further analyzed by XPS. By comparing the high-resolution Fe 2p and Ni 2p XPS spectra of NiFe-CNT and Li_2_S_6_/NiFe-CNT (Figure 6c,d), it can be observed that the Fe 2p_3/2_ peak shifts from 712.5 to 712.7 eV and the Ni 2p_3/2_ peak shifts from 855.7 to 855.9 eV after the adsorption of Li_2_S_6_. This suggests strong interactions between Fe atoms and sulfur as well as Ni atoms and sulfur [34,35]. Furthermore, according to the literature [7], DFT calculations predict that the -OH functional groups on the surface of LDHs may form hydrogen bonds with the S atom in lithium polysulfides. Therefore, NiFe-CNT demonstrates excellent adsorption capability for lithium polysulfides. 

## 4. Conclusions

In summary, we synthesized NiFe-LDHs-modified CNTs and used them as the sulfur host with the purpose of boosting the field of lithium–sulfur batteries. Compared with pure NiFe-LDHs, the incorporation of CNTs into NiFe-LDHs led to a significant enhancement in the electrochemical performance of sulfur. This improvement can be attributed to the well-dispersed NiFe-LDH nanosheets on CNTs, resulting in a larger contact area between sulfur and NiFe-LDHs. Consequently, NiFe-CNT exhibits strong adsorption for lithium polysulfides and effectively promotes their conversion, thereby suppressing the shuttle effect. In addition, the cross-linked carbon nanotubes (CNTs) form a conductive network that can enhance both the electron conductivity of NiFe-CNT@S and the diffusion of lithium ions. This study demonstrates that by combining LDH nanosheets with conductive carbon materials, their performance as sulfur hosts can be significantly enhanced. 

## Figures and Tables

**Table 1:** Summary of the electrochemical performance of NiFe-CNT@S and other reported LDH-based sulfur materials.

Samples	Cycle Number	Current Density	Initial Capacity	Final Capacity	Capacity Decay Rate per Cycle	Ref.
Co-Fe LDH@S	500	1 C	755	387	0.098%	[30]
S/LDH/rGO	200	1 C	730	394	0.230%	[31]
S@Ni/Fe LDH	1000	1 C	844	501	0.041%	[32]
S/NiCo-LDH	500	1 C	609	475	0.044%	[33]
NiFe-CNT@S	500	2 C	1010	876	0.026%	This work

## Data Availability

The data used to support the findings of this study are included within the article.

## Supplementary Materials

The following supporting information can be downloaded at https://www.mdpi.com/article/10.3390/nano14030272/s1, Figure S1: Raman spectra of NiFe-CNT and CNTs; Figure S2: SEM-EDX elemental mappings of NiFe-LDH@S; Figure S3: Randles equivalent circuit for NiFe-LDH@S and NiFe-CNT@S.
