# Supplementary material for: Fabrication of NiFe-LDHs Modified Carbon Nanotubes as the High-Performance Sulfur Host for Lithium–Sulfur Batteries
Source: Nanomaterials (Basel). 2024 Jan 27;14(3):272. doi: 10.3390/nano14030272 (PMC10856954; doi:10.3390/nano14030272)
Supplement: Supplementary file 1 [file nanomaterials-14-00272-s001.zip › nanomaterials-2821451-supplementary.pdf]

Supporting Information

Fabrication of NiFe-LDHs Modified Carbon Nanotubes as the High-Performance Sulfur Host for Lithium–Sulfur Batteries

Lingwei Zhang [1,2], Runlan Li [1] and Wenbo Yue [1,*]

[1] Beijing Key Laboratory of Energy Conversion and Storage Materials, College of Chemistry, Beijing Normal University, Beijing 100875, P. R. China

[2] College of Chemistry, Xi'an Jiaotong University, Xi'an 710049, China

*Corresponding authors:

Yue WB, Tel: +86 (0)10 58804229, E-mail: wbyue@bnu.edu.cn

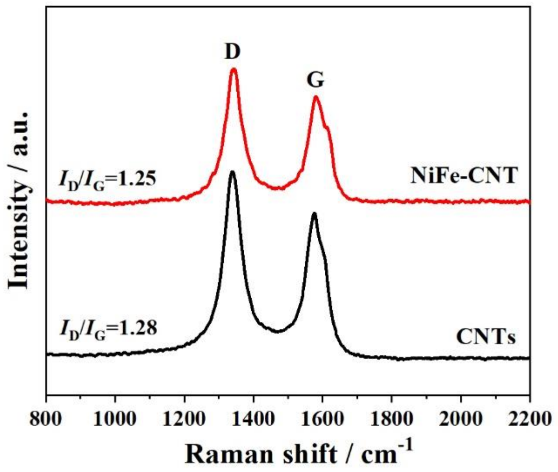

Figure S1. Raman spectra of NiFe-CNT and CNTs.

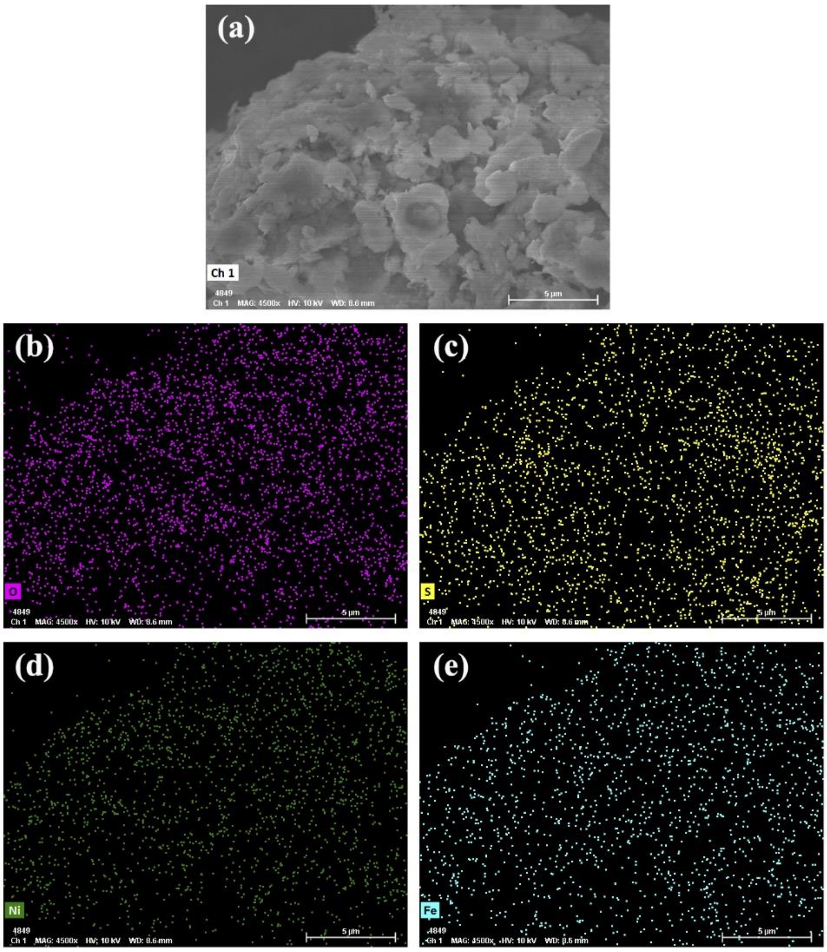

Figure S2. (a) SEM image of NiFe-LDH@S and EDX elemental mappings of (b) O, (c) S, (d) Ni and (e) Fe.

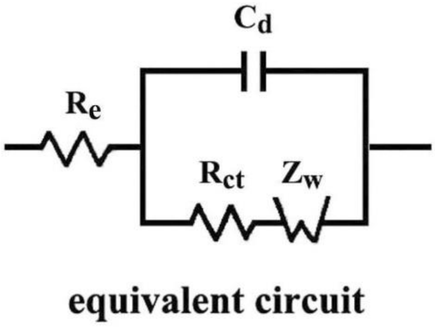

Figure S3. Randles equivalent circuit for NiFe-LDH@S and NiFe-CNT@S.
